# Validity of a Four-Item Household Water Insecurity Experiences Scale for Assessing Water Issues Related to Health and Well-Being

**DOI:** 10.4269/ajtmh.20-0417

**Published:** 2020-10-26

**Authors:** Sera L. Young, Joshua D. Miller, Edward A. Frongillo, Godfred O. Boateng, Zeina Jamaluddine, Torsten B. Neilands, and the HWISE Research Coordination Network

**Affiliations:** 1 Department of Anthropology, Northwestern University, Evanston, Illinois;; 2 Institute for Policy Research, Northwestern University, Evanston, Illinois;; 3 Department of Health Promotion, Education, and Behavior, University of South Carolina, Columbia, South Carolina;; 4 Department of Kinesiology, College of Nursing and Health Innovation, University of Texas at Arlington, Arlington, Texas;; 5 Center for Research on Population and Health, American University of Beirut, Beirut, Lebanon;; 6 School of Medicine, University of California San Francisco, San Francisco, California

## Abstract

We sought to determine whether a shortened version of the 12-item Household Water Insecurity Experiences (HWISE) Scale, which measures water insecurity equivalently in low- and middle-income countries (LMICs), is valid for broad use. Using data from 9,261 households in 25 LMICs, subsets of candidate items were evaluated on their predictive accuracy, criterion validity, and sensitivity–specificity. A subset with items assessing “worry,” “changing plans,” “limited drinking water,” and “inability to wash hands” because of problems with water (range: 0–12) were highly correlated with full HWISE Scale scores (correlation coefficient: 0.949–0.980) and introduced minimal additional error (root mean square error: 2.13–2.68). Criterion validity was demonstrated, and a cut point of ≥ 4 correctly classified more than 91% of households as water secure or insecure. The brief HWISE-4 can be used in LMICs to inform decisions about how to most effectively target resources and evaluate public health interventions.

## INTRODUCTION

Water is fundamental to physical, nutritional, and psychosocial well-being.^1,2^ Issues with water availability, accessibility, reliability, and use globally present significant risks to health and development.^3–5^ The concept of household water insecurity, which considers each of these constructs concurrently, is a powerful way to understand how water impacts well-being.^6^

Quantification of household water insecurity is important for assessing its prevalence, identifying inequities (e.g., by gender and race),^7^ and understanding its relationships with other phenomena, including the impact of public health interventions. Although site-specific scales for measuring household water insecurity have been created, they are not appropriate for cross-cultural comparisons.^6^ The 12-item Household Water Insecurity Experiences (HWISE) Scale was therefore developed to measure water insecurity comparably across low- and middle-income countries (LMICs).^8^ Its use has revealed that water insecurity is associated with myriad outcomes, including greater food insecurity,^8^ higher stress,^8^ and greater odds of diarrhea.^9^

Although the HWISE Scale is relatively brief^10^ and is preferred for measuring household water insecurity, the estimated three minutes required is too burdensome or costly for many surveys. Nationally representative surveys that are instrumental for informing global and public health policies have stringent criteria for retaining or adding new items.^11,12^ Items must provide novel and actionable data while introducing minimal time burdens for enumerators and participants. Such issues are particularly salient in emergency contexts where rapid assessment is critical and/or when telephone interviewing is used. Therefore, to promote uptake of this tool by agencies that prioritize shorter survey length and to generate a greater understanding of water insecurity, we sought to determine whether a shortened version of the HWISE Scale, the HWISE-4 Scale, is valid for broad use.

## METHODS

### Criteria and analytic approach.

The original HWISE Scale was designed using classical test theory to measure all components of household water insecurity in the prior 4 weeks. Responses to each item— “never” (scored as 0), “rarely” (1–2 times, scored as 1), “sometimes” (3–10 times, scored as 2), and “often or always” (> 10 times, scored as 3)—are summed to create a continuous score (range: 0–36). Here, we sought to identify a brief subset of these items (i.e., five or fewer) that would take less than 1 minute to administer and meet four criteria: be equivalent across contexts, be easily answerable, capture a range of severity, and assess key constructs (availability, access, use, and reliability) of water insecurity.

Given that equivalence was previously established for all 12 items using alignment optimization, no items were eliminated because of measurement non-invariance.^8^ We used cognitive interviews and debriefing notes from scale administrators to identify items that required additional prompts to answer. We then identified which constructs of water insecurity each item captured and classified each item’s relative severity using Rasch analysis.^8^ To select between items with similar severity scores, we chose those that were more strongly correlated with full HWISE Scale scores.

We evaluated candidate subsets based on their predictive accuracy. To do this, we regressed full HWISE Scale scores on each subset, controlling for site as a fixed effect. We then compared the relative additional error introduced when estimating household water insecurity scores using each subset by comparing root mean square errors.

Subsequently, criterion validity of candidate subsets was assessed using the same criteria applied when developing the original HWISE Scale.^8^ In brief, we first tested predictive validity, that is, the extent to which a measure predicts the answers to some other question or a measurement to which it ought to be related.^13^ We did this by regressing food insecurity, perceived stress, satisfaction with water situation, and perceived water standing in the community on subset scores. Convergent validity, or the extent to which a construct measured in different ways yields similar results,^14^ was assessed by examining the association between subset scores and time to drinking water source. Discriminant validity, or the extent to which a measure is novel and not simply a reflection of some other construct,^15^ was tested using differentiation between “known groups”; specifically, we compared whether subset scores differed between those who did and did not report injury during water acquisition. All models accounted for clustering by site.

Finally, we sought to determine if there was a cut point that could accurately distinguish between water-secure and water-insecure households comparable to the provisional cut point for the full HWISE Scale (scores ≥ 12).^8^ We did this by creating receiver-operating characteristic curves and comparing the proportion of correctly specified households for each cut point. All analyses were completed using Stata 14.0 (StataCorp, College Station, TX).

### Study design.

Data were drawn from the 28 sites in 23 countries included in the original HWISE Scale development study (Supplemental Table S1). Data collection is described in greater detail elsewhere.^8,16^ Trained enumerators collected survey data in a consistent manner across sites. Data collection occurred in two waves between 2017 and 2018 as the survey was refined, such that only wave 2 sites have full HWISE Scale scores.^8^ Data from Demographic and Health Survey sites in Dhaka and Chakaria, Bangladesh, were also added to this dataset; these data were collected following wave 2 study protocol^16^ but were not published in the validation study because of delays in data collection. Households were included if they had sufficient data (i.e., responses to all 12 items) for calculating complete HWISE Scale scores (wave 2, *n* = 3,293) or HWISE-4 subset scores (wave 1, *n* = 4,058).

To determine if our findings were replicable in settings not associated with scale development, we also used data from Oxfam Great Britain’s 2019 Effectiveness Reviews in North Kivu, Democratic Republic of Congo (*n* = 988), and Lusaka, Zambia (*n* = 922), where the HWISE Scale was implemented in cross-sectional surveys.^9^ Study activities were approved by all relevant ethical review boards, as reported elsewhere (Supplemental Table S1).^9,16^

## RESULTS

Four of the original 12 items were identified as sometimes being slightly more difficult to answer, and thus were not considered for inclusion (Supplemental Table S2). Two pairs of items had similarly high (“wash hands” and “thirsty”) or low (“worry” and “supply interrupted”) severity scores based on the Rasch analysis; we chose the item from each that was more strongly correlated with HWISE Scale scores (“worry” and “wash hands”). Of the four remaining candidate items, “wash body” was excluded because it was conceptually similar to “wash hands.” With the remaining items, a subset of four items about “worry,” “changing plans,” “limited drinking water,” and “washing hands” and a subset of five items adding “no water whatsoever,” were created.

**Figure 1.**
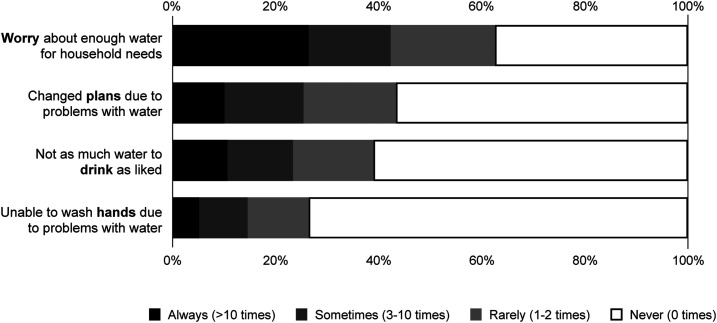
Frequency of affirmation of Household Water Insecurity Experiences (HWISE) items included in the HWISE-4 Scale, across 13 HWISE wave 2 study sites in 12 low- and middle-income countries (*n* = 3,293).^8^

Both subsets were positively associated with full HWISE Scale scores. Root mean square errors ranged from 1.56 to 2.68 across the two subsets and three samples (Supplemental Table S3). The subset of five items introduced less additional error in estimating water insecurity scores than the subset of four items, but the difference was small (e.g., 2.08 versus 2.45). Given that brevity was a primary aim, we proceeded with the four-item subset (range: 0–12) (Table 1, Figure 1).

**Table 1:** The Household Water Insecurity Experiences 4-item (HWISE-4) short form

Label	Item*
Worry	In the last 4 weeks, how frequently did you or anyone in your household worry you would not have enough water for all of your household needs?
Plans	In the last 4 weeks, how frequently have you or anyone in your household had to change schedules or plans because of problems with your water situation? (Activities that may have been interrupted include caring for others, doing household chores, agricultural work, income-generating activities, and sleeping)
Drink	In the last 4 weeks, how frequently has there not been as much water to drink as you would like for you or anyone in your household?
Hands	In the last 4 weeks, how frequently have you or anyone in your household had to go without washing hands after dirty activities (e.g., defecating, changing diapers, cleaning animal dung) because of problems with water?

* Responses to items are as follows: never (0 times), rarely (1–2 times), sometimes (3–10 times), often (11–20 times), always (more than 20 times), do not know, and not applicable/I do not have this. Never is scored as 0, rarely is scored as 1, sometimes is scored as 2, and often/always is scored as 3. Responses are added together for a summative score. A score of *≥* 4 indicates household water insecurity. Items are drawn from ref. 8.

Criterion validity of the four-item subset, that is, predictive, convergent, and discriminant validity, was established using data from the wave 2 HWISE study sites (Supplemental Table S4). For example, for every 3.0 points higher on the HWISE-4 Scale, households in HWISE wave 2 study sites were expected to score 2.4 (95% CI: 1.65–3.21) points higher on the Household Food Insecurity Access Scale.

**Figure 2.**
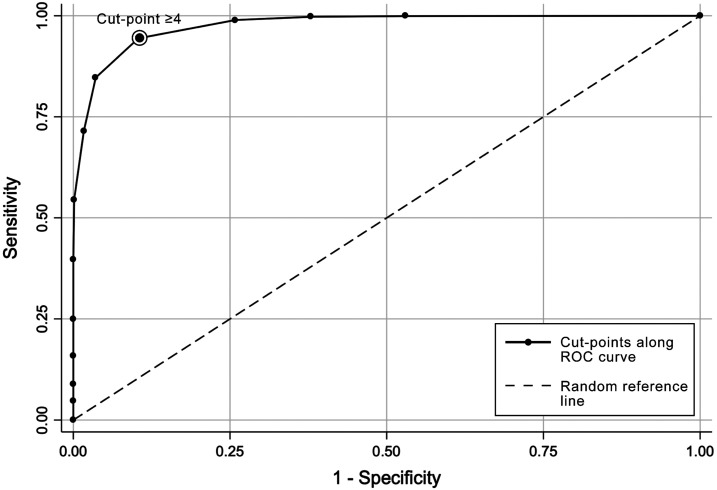
Sensitivity and specificity of different cut points for classifying household water insecurity using the Household Water Insecurity Experiences (HWISE)-4 Scale. A cut point of ≥ 4 accurately classifies households as water secure or insecure with high specificity and sensitivity, as demonstrated using data collected in 13 HWISE wave 2 study sites (*n* = 3,293).

We then assessed at which cut point the four-item subset most accurately classified households as water secure or insecure compared with the HWISE Scale. A cut point of ≥ 4 correctly classified > 91% of households across all study populations (Supplemental Table S5) with high sensitivity and specificity (Figure 2).

## DISCUSSION

The 4-item HWISE-4 short form, which takes approximately one minute to administer, captures a range of experiences and severity across salient constructs. The HWISE-4 Scale accurately classified household water insecurity with little additional error compared with the full HWISE Scale.

This study used diverse sites, rigorous data collection and analytic methods, and best practices in scale development. Furthermore, findings about the HWISE Scale were replicated in data collected by an external group.

We encourage the use of the full 12-item HWISE Scale because it provides a more comprehensive and accurate estimation of water insecurity. When resources or survey time are limited, however, the HWISE-4 Scale can be used to assess water insecurity with minimal additional error, providing valuable information about whether water access, use, and/or reliability is problematic.

## CONCLUSION

The HWISE-4 Scale can be used in LMICs to quickly identify water-insecure households, inform decisions about how to most effectively target resources, and evaluate public health interventions. For instance, the HWISE-4 Scale can determine which households are experiencing water-related issues that prevent regular handwashing, which is important for preventing transmission of many infectious diseases. In sum, the HWISE-4 Scale is a valid tool for measuring household water insecurity when resources are constrained.

## Supplemental tables

Supplemental materials
