# Supplementary material for: Validity of a Four-Item Household Water Insecurity Experiences Scale for Assessing Water Issues Related to Health and Well-Being
Source: Am J Trop Med Hyg. 2020 Oct 26;104(1):391–4. doi: 10.4269/ajtmh.20-0417 (PMC7790094; doi:10.4269/ajtmh.20-0417)
Supplement: Supplementary file 1 [file tpmd200417.SD1.pdf]

Supplemental Table S1. Characteristics of the sites included in HWISE short form analyses, by data source.

Data source	Site	Season of data collection	Proportion of female respondents	HWISE Scale score*, mean(sd)	Data available for short form analyses†		
					Accuracy	Validity	Sensitivity-specificty
HWISE scale development study, wave 1	Mérida, Mexico (n=247)	Dry season	62.8	–		X	
	Acatenango, Guatemala (n=95)	Dry season	92.5	–		X	
	Honda, Colombia (n=170)	Rainy season	64.7	–		X	
	Ceará, Brazil (n=201)	Neither rainy nor dry season	71.6	–		X	
	Accra, Ghana (n=227)	Rainy season	78.4	–		X	
	Lagos, Nigeria (n=235)	Rainy season	73.0	–		X	
	Kahemba, Democratic Republic of Congo (n=390)	Dry season	65.9	–		X	
	Bahir Dar, Ethiopia (n=253)	Rainy season	100	–		X	
	Kampala, Uganda (n=236)	Dry season	68.6	–		X	
	Arua, Uganda (n=242)	Rainy season	85.1	–		X	
	Kisumu, Kenya (n=247)	Neither rainy nor dry season	81.3	–		X	
	Singida, Tanzania (n=563)	Dry season	56.7	–		X	
	Lilongwe, Malawi (n=297)	Neither rainy nor dry season	86.9	–		X	
	Dushanbe, Tajikistan (n=222)	Dry season	73.4	–		X	
	Kathmandu, Nepal (n=259)	Rainy season	71.8	–		X	
	Upolu, Samoa (n=174)	Across multiple seasons	57.0	–		X	
HWISE scale development study, wave 2	Torreón, Mexico (n=239)	Dry season	72.8	8.6 (8.4)	X	X	X
	Gressier, Haiti (n=280)	Dry season	98.6	9.8 (9.1)	X	X	X
	San Borja, Bolivia (n=171)	Dry season	62.8	17.5 (7.9)	X	X	X
	Chiquimula, Guatemala (n=286)	Dry season	85.7	5.2 (5.3)	X	X	X
	Cartagena, Colombia (n=218)	Dry season	70.6	20.9 (7.5)	X	X	X
	Morogoro, Tanzania (n=256)	Rainy season	78.1	4.2 (4.8)	X	X	X
	Sistan & Balochistan, Iran (n=132)	Rainy season	99.2	6.0 (6.5)	X	X	X

	Beirut, Lebanon (n=544)	Rainy season	64.2	7.1 (7.0)	X	X	X
	Punjab, Pakistan (n=47)	Dry season	76.6	20.4 (5.9)	X	X	X
	Pune, India (n=171)	Across multiple seasons	100	1.6 (4.2)	X	X	X
	Rajasthan, India (n=208)	Dry season	27.4	13.9 (7.4)	X	X	X
	Dhaka & Chakaria, Bangladesh (n=473)[‡]	Rainy season	97.0	6.9 (8.0)	X	X	X
	Labuan Bajo, Indonesia (n=268)	Dry season	44.4	13.8 (7.7)	X	X	X
Oxfam Great Britain Effectiveness Reviews	North Kivu, Democratic Republic of Congo (n=988)	Dry season	48.6	10.7 (9.9)	X		X
	Lusaka, Zambia (n=922)	Dry season	52.9	9.9 (10.8)	X		X

[*]HWISE Scale score range: 0-36
[†]*Accuracy*: data needed to compare subset score to full HWISE Scale scores; *validity*: data needed to assess relationships between subset scores and food insecurity, perceived stress, satisfaction with water situation, perceived water standing in the community, time to water source, and injury while fetching water; *sensitivity-specificity*: data needed to compare the proportion of correctly specified households, as determined using full HWISE Scale scores, for each cut-point in the candidate subset
[‡]Data collected following HWISE study protocol but not published in validation manuscript

Supplemental Table S2. Characteristics of candidate items for the HWISE short form, ordered by Rasch severity scores for HWISE wave 2 study sites.

HWISE Scale items	Issues noted by implementers	Mean severity score across sites[†]	Correlation coefficient[‡]	Water insecurity constructs			
				Availability	Accessibility	Reliability	Use
**Worry about enough water for all household needs**		1.82	0.730	X	X	X	X
Water supply interrupted		2.18	0.697		X	X	
Unable to wash clothes	Some respondents washed their clothes off premises and required additional guidance to answer	3.00	0.825				X
Upset or angry about water situation	This was considered a politically sensitive question in some settings and required additional prompting	4.27	0.705	X	X	X	X
**Changed plans due to water situation**		5.27	0.778		X	X	
**Not as much water to drink as liked**		6.45	0.758				X
Unable to wash body		7.45	0.774				X
Changed what foods were prepared	Male respondents may underreport this if a female household member primarily determines which foods are prepared	7.55	0.757				X
**No water in the household whatsoever**		8.73	0.691				X
Felt ashamed about water situation	This was considered a sensitive question in some settings and required additional prompting	9.73	0.577		X	X	X
**Unable to wash hands**		10.36	0.727				X
Went to sleep thirsty		11.18	0.538				X

[†]Rasch severity scores only available for the 12 wave 2 sites included in the original HWISE Scale validation study
[‡]Calculated using data from all 13 HWISE wave 2 study sites
Bolded items are those included in the candidate 4- and 5-item subsets.

Supplemental Table S3. Linear regressions of full HWISE Scale scores on two candidate subsets of items and their internal consistency, by study population and controlling for site.

	Subset of four items† (range: 0-12)					Subset of five items‡ (range: 0-15)				
	**β**	***p*-value**	**RMSE**	**Correlation coefficient**	**Cronbach's alpha**	**β**	***p*-value**	**RMSE**	**Correlation coefficient**	**Cronbach's alpha**
**HWISE wave 1 study sites** (n=4058)	–	–	–	–	0.713	–	–	–	–	0.762
**HWISE wave 2 study sites** (n=3293)	2.45	<0.001	2.68	0.949	0.789	2.08	<0.001	2.42	0.959	0.822
**North Kivu, DRC** (n=988)	2.91	<0.001	2.13	0.980	0.925	2.36	<0.001	1.56	0.990	0.938
**Lusaka, Zambia** (n=922)	2.88	<0.001	2.48	0.968	0.851	2.34	<0.001	1.84	0.983	0.885

†Subset of four items: worry, change plans, not as much to drink as liked, and unable to wash hands
‡Subset of five items: worry, change plans, not as much to drink as liked, unable to wash hands, and no water whatsoever
RMSE: root mean square error, i.e., standard deviation of residuals

Supplemental Table S4. Tests of the criterion validity of the four-item HWISE subset in (A) 16 HWISE wave 1 study sites in 14 low- and middle-income countries and (B) 13 HWISE wave 2 study sites in 12 low- and middle-income countries, using random coefficient regression models controlling for site.

(A)

	Coefficient (95% CI)	RMSE	ICC
**Predictive validity**			
4-item Perceived Stress Scale score (0-16) (n=3824)	0.25 (0.17, 0.33)***	2.56	0.19
Household Food Insecurity Access score (0-27) (n=3759)	0.96 (0.78, 1.13)***	4.52	0.27
**Convergent validity**			
Time (minutes) to water source (n=3719)	0.02 (0.02, 0.03)***	1.96	0.28
**Discriminant validity**			
If injured while fetching water (n=2505)	1.41 (0.93, 1.88)***	1.88	0.38

* *p*<0.05; ** *p*<0.01; ****p*<0.0001
†Four items: worry, change plans, not as much to drink as liked, and unable to wash hands
RMSE: root mean square error, i.e., standard deviation of residuals
ICC: intraclass correlation

(B)

	Coefficient (95% CI)	RMSE	ICC
**Predictive validity**			
Satisfaction with water situation (n=3291)	-0.17 (-0.20, -0.15)***	1.12	0.26
Perceived water standing in community (n=3208)	0.36 (0.26, 0.45)***	2.33	0.12
4-item Perceived Stress Scale score (0-16) (n=3227)	0.12 (0.04, 0.20)**	2.41	0.13
Household Food Insecurity Access score (0-27) (n=3142)	0.80 (0.55, 1.07)***	5.57	0.27
**Convergent validity**			
Time (minutes) to water source (n=2959)	0.02 (0.01, 0.03)***	2.63	0.38
**Discriminant validity**			
If injured while fetching water (n=2696)	1.50 (0.64, 2.37)**	2.73	0.38

* *p*<0.05; ** *p*<0.01; ****p*<0.0001
†Four items: worry, change plans, not as much to drink as liked, and unable to wash hands
RMSE: root mean square error, i.e., standard deviation of residuals
ICC: intraclass correlation

Supplemental Table S5. Proportion of households correctly classified as being water secure or insecure on the basis of the cut-point of ≥12 in the full HWISE scale (range: 0-36), by selected cut-points for the four-item HWISE subset (range: 0-12).

	**>=3**	**>=4**	**>=5**
**HWISE wave 2 study sites** (n=3293)	82.75	91.28	92.35
**North Kivu, DRC** (n=988)	91.80	93.42	88.06
**Lusaka, Zambia** (n=922)	84.71	91.65	89.59
